# Orbital Cellulitis and Frontal Epicranial Empyema Secondary to Pansinusitis: A Case Report

**DOI:** 10.7759/cureus.50908

**Published:** 2023-12-21

**Authors:** Manuel Tousidonis, Marta Benito-Anguita, Angela Sada-Urmeneta, Juan-Jose Verdaguer-Martin, Fernando Ruiz-Juretschke

**Affiliations:** 1 Oral and Maxillofacial Surgery, Gregorio Marañón University Hospital, Madrid, ESP; 2 Oral and Maxillofacial Surgery, Gregorio Marañon University Hospital, Madrid, ESP; 3 Neurosurgery, Gregorio Marañon University Hospital, Madrid, ESP

**Keywords:** intracranial epidural abscess, infectious disease, intracranial complications, chronic sinusitis, orbital cellulitis

## Abstract

Orbital cellulitis is a relatively uncommon complication of sinusitis. Its association with intracranial complications is rare. We present the case of a 36-year-old patient with no associated risk factors who presented with a four-day history of headache, periorbital inflammation, suppuration, and necrosis. A computed tomography (CT) scan revealed a frontal epidural abscess and signs of chronic pansinusitis. This case highlights the importance of maintaining a high index of suspicion for complications of this condition and the necessity of a multidisciplinary approach in managing this rare complication.

## Introduction

Orbital cellulitis, also known as postseptal cellulitis, is a rare condition. It is defined as an acute infectious inflammation of the orbital and periorbital soft tissues, including the skin, periorbital subcutaneous tissue [1], extraocular muscles, neurovascular structures, and paranasal sinuses [2]. It presents with edema, erythema, and swelling of the eyelid. Unlike preseptal cellulitis, it can cause pain with eye movements, decreased visual acuity, proptosis, ophthalmoplegia, afferent pupillary defect [3], and even intracranial involvement in cases of unfavorable progression [4,5]. Although both conditions can coexist [2], it is important to differentiate them because the treatment strategy is different, with postseptal cellulitis requiring early intravenous antibiotic therapy and even surgical drainage [2,6].

In this case report, we present the case of a patient with orbital cellulitis and intracranial extension secondary to pansinusitis. Awareness of the possible spread of orbital cellulitis to the brain is extremely important, so vigorous treatment may start as early as possible. The patient required urgent multidisciplinary surgical management in addition to intensive intravenous antibiotic therapy, with a favorable outcome.

## Case presentation

A 36-year-old male with a history of childhood sinusitis presented to the emergency department with a four-day history of headache, swelling, purulent drainage, and periorbital necrosis.

**Figure 1:**
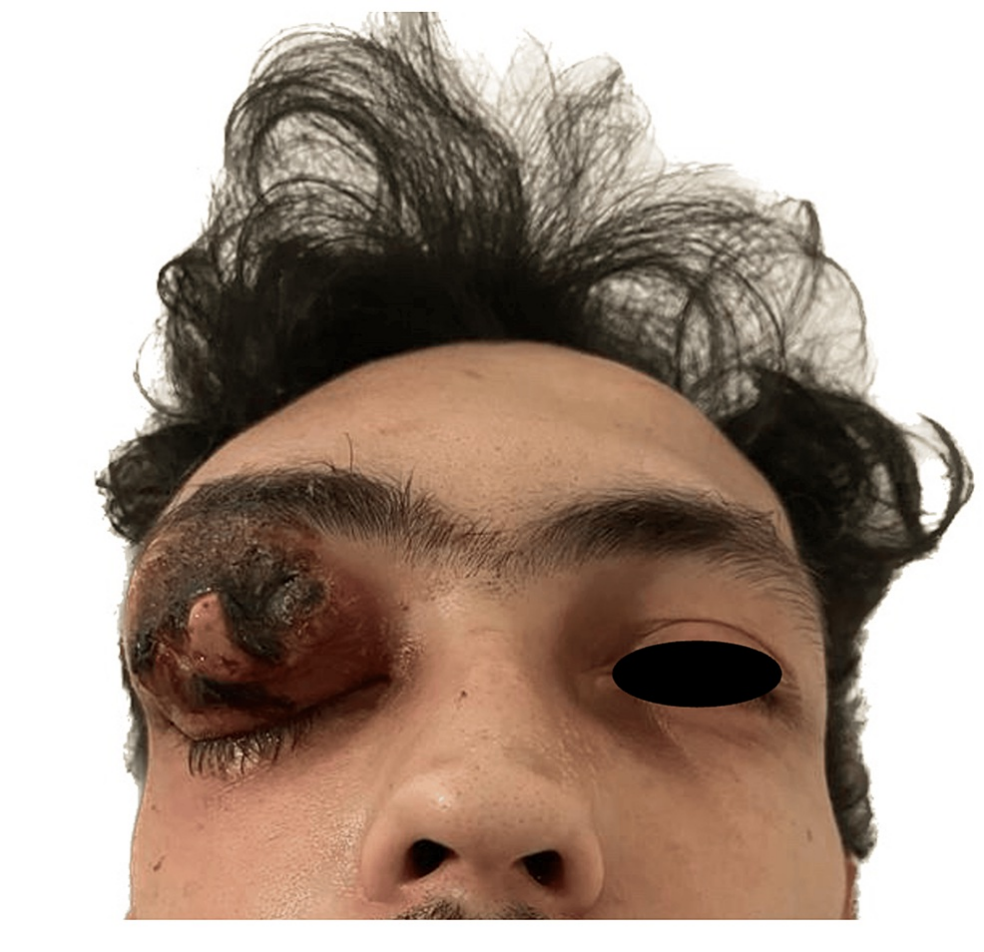
Orbital cellulitis and necrosis Clinical examination of the patient at the emergency department, in which a necrotic lesion with edema, erythema, and proptosis of the upper right eyelid can be observed.

On physical examination, he was afebrile and hemodynamically stable. He had edema, erythema, and proptosis of the right upper eyelid with extension to the ipsilateral frontal region and necrotic skin lesions with purulent drainage (Figure 1). Visual acuity and extrinsic eye movements remained intact.

**Figure 2:**
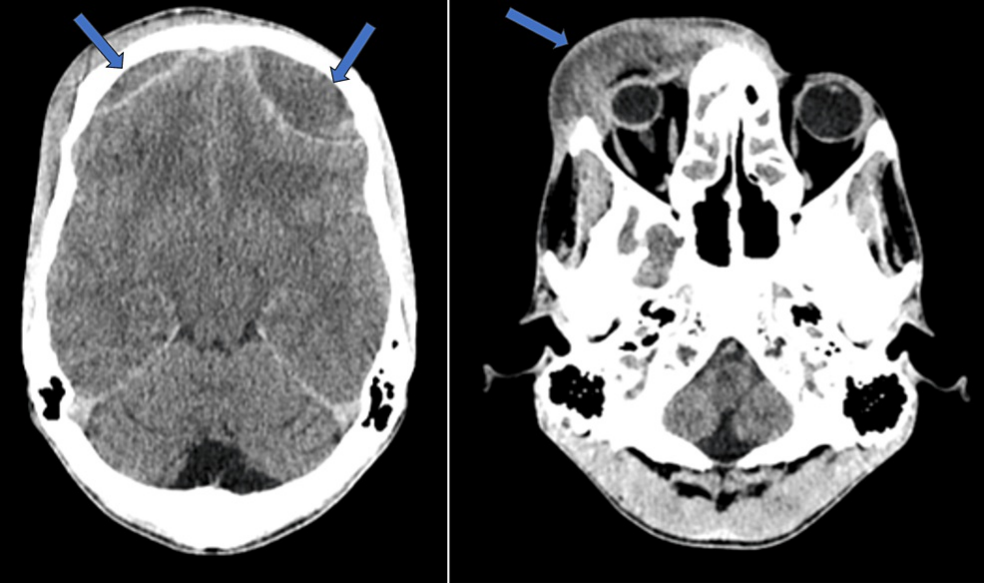
Orbital cellulitis and empyema in CT In the CT scan, collections are visualized in the preseptal, frontal, and right nasal dorsum regions, measuring approximately 7 × 1.7 cm in maximum axial and 5.7 cm in the longitudinal axis, extending into the intraorbital extraconal area adjacent to the orbit roof. The eyeball, extraocular orbital muscles, and optic nerve showed no abnormalities. Additionally, there was a bilateral epicranial intracranial collection with a maximum thickness of 2.4 cm, causing a mass effect on adjacent parenchyma and sulcus obliteration, associated with superior longitudinal sinus thrombosis. The anterior wall of the right frontal sinus showed signs of osteomyelitis. Furthermore, signs of chronic sinusitis were detected in the frontal sinuses, ethmoidal cells, and maxillary sinuses.

Laboratory analysis showed leukocytosis (13,000 cells/mm^3^). Blood cultures and rapid streptococcal group A test were negative. Computed tomography (CT) revealed inflammatory changes with abscess collections in the preseptal, frontal, and right nasal dorsum regions with extraconal orbital extension adjacent to the orbit roof. Additionally, there was a bilateral epicranial frontal collection with superior longitudinal sinus thrombosis. Signs of chronic sinusitis were observed in the frontal, ethmoid, and maxillary sinuses (Figure 2).

**Figure 3:**
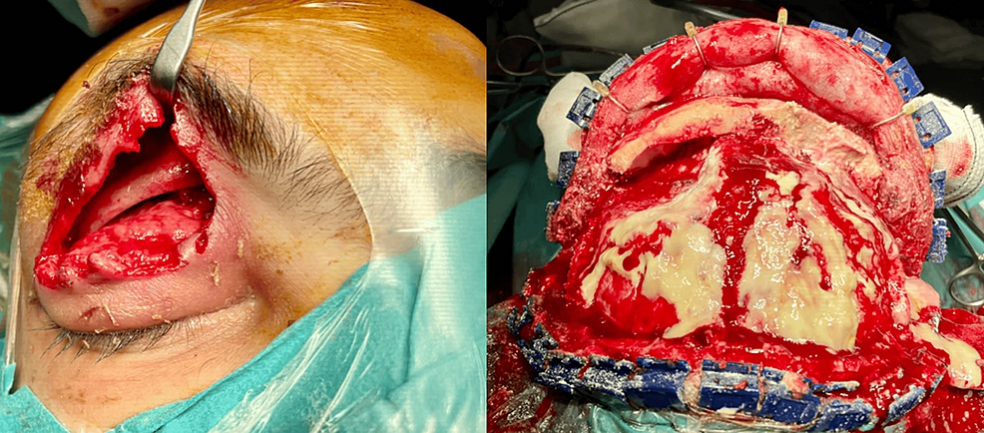
Surgical treatment Surgical drainage was performed through a transseptal approach for the orbital collection that extended subperiosteally through the orbit roof and frontal bone. Frontal epicranial collections were drained through a bicoronal craniotomy without a dural opening.

The patient was treated with broad-spectrum intravenous antibiotic therapy. A multidisciplinary surgical approach was performed, involving maxillofacial surgery, neurosurgery, and otorhinolaryngology (Figure 3).

The collection with orbital extension was drained through a transseptal approach. A bicoronal craniotomy was also necessary to drain the epicranial frontal collections (Videos 1, 2).

**Video 1:** Orbital cellulitis and frontal epicranial empyema secondary to pansinusitis: a case report

**Video 2:** Orbital cellulitis and frontal epicranial empyema secondary to pansinusitis: a case report

Finally, a bilateral endoscopic anterior ethmoidectomy was performed. Cultures were positive for *Streptococcus viridans*. After two weeks of hospitalization, the patient was discharged with one more week of oral antibiotic therapy.

**Figure 4:**
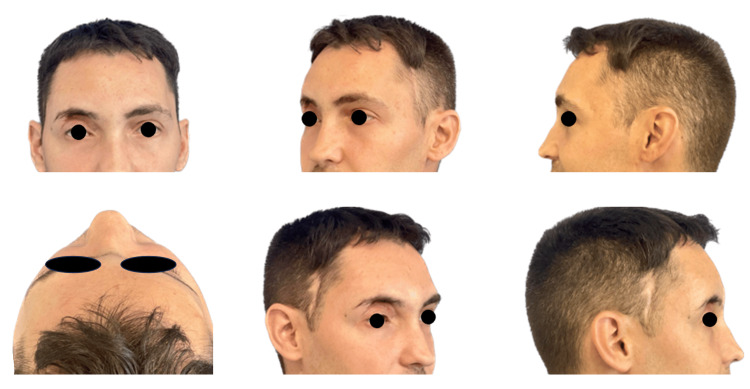
Appearance of the patient one year after the intervention without having performed surgery for aesthetic sequelae due to patient refusal

During follow-up, one year later, the patient developed chronic osteomyelitis with remodeling of the right frontal bone (Figure 4), with no progression or impact on daily activities. Therefore, the patient opted to avoid further surgery and continued with conservative treatment.

## Discussion

Preseptal cellulitis is an infection of the skin and soft tissues anterior to the orbital septum. Postseptal cellulitis occurs when the infection extends beyond the orbital septum [3]. Both conditions are more common in children [4,6]. Immunocompromised individuals are at higher risk [2]. Most cases are caused by the extension of sinusitis, but they can exceptionally result from odontogenic infection, trauma, or periocular surgeries [4,7]. Although the prognosis is generally favorable, orbital complications have been reported in 1.7-7% of cases [5-9].

The orbit is a conical structure with walls in proximity to the paranasal sinuses. Venous drainage from the orbit, ethmoid sinus, maxillary sinus, skin, eyelid, and periorbital tissue converges into the cavernous sinus. This venous system lacks valves and has anastomoses with the ophthalmic veins. Therefore, paranasal infections can progress to the orbit and intracranial structures [6,9].

The diagnosis of preseptal cellulitis is clinical, characterized by eyelid edema, erythema, and redness [3]. Imaging techniques such as CT or magnetic resonance imaging (MRI) should be considered in cases of ophthalmoplegia, proptosis, decreased visual acuity, pain with eye movements, suspicion of intracranial involvement, or failure of conservative treatment [1,5].

Necrotizing fasciitis should be considered as a cause of skin and subcutaneous tissue inflammation that extends to affect different fascial planes. Only 10% of cases involve the head and neck region. Beta-hemolytic group A Streptococcus is the most common microorganism involved [10], with no reported cases of necrotizing fasciitis caused by *S. viridans*.

Orbital cellulitis should be treated with early intravenous antibiotic therapy and surgical intervention if there is the presence of abscesses [6], failure of conservative treatment, suspicion of optic nerve involvement [11], or intracranial extension [12]. Negative pressure therapy has been suggested by some authors to accelerate the healing process and reduce hospital stays [6].

Blood cultures and cultures of drained material should be obtained, although blood cultures are often negative [1]. The most frequently isolated microorganisms are staphylococci and streptococci [1,4]. Some studies suggest that community-acquired methicillin-resistant *Staphylococcus aureus* may be an emerging cause of orbital cellulitis [3,13].

The most common complication is subperiosteal abscess [4]. Less frequently, orbital abscesses can occur, leading to proptosis, ophthalmoplegia, or optic nerve ischemia [9]. In severe cases, extraorbital extension with the formation of brain abscesses [11], frontal bone osteomyelitis [14], meningitis, septic cavernous sinus thrombosis [6,11], and sepsis is possible [3]. Therefore, early diagnosis and treatment are crucial to prevent these serious complications [1].

## Conclusions

Orbital cellulitis as a complication of sinusitis is a very rare condition, but its consequences can be severe and result in significant sequelae. Therefore, early diagnosis and multidisciplinary early treatment are essential. In most cases, the prognosis is favorable.

## References

[1] Ibarrondo Pastrana J, Lorente Guerrero J, Serra Carreras J, García López M, Quesada Martínez JL, Quesada Marín P, Perelló Scherdel E (2002). Orbital cellulitis: review and presentation of 20 cases. An Otorrinolaringol Ibero Am.

[2] Lim LT, Miller D, Ah-Kee EY, Ferguson A (2015). Preseptal cellulitis or orbital cellulitis?. West Indian Med J.

[3] Pandian DG, Babu RK, Chaitra A, Anjali A, Rao VA, Srinivasan R (2011). Nine years' review on preseptal and orbital cellulitis and emergence of community-acquired methicillin-resistant Staphylococcus aureus in a tertiary hospital in India. Indian J Ophthalmol.

[4] Stead TG, Retana A, Houck J, Sleigh BC, Ganti L (2019). Preseptal and postseptal orbital cellulitis of odontogenic origin. Cureus.

[5] Al-Madani MV, Khatatbeh AE, Rawashdeh RZ, Al-Khtoum NF, Shawagfeh NR (2013). The prevalence of orbital complications among children and adults with acute rhinosinusitis. Braz J Otorhinolaryngol.

[6] Fernández JB, Fernández MM (2014). Celulitis preseptal y orbitaria. Pediatr Integr.

[7] Contreras-Ruiz J, Ramos-Cadena A, Solis-Arias P (2015). Negative pressure wound therapy in preseptal orbital cellulitis complicated with necrotizing fasciitis and preseptal abscess. Ophthalmic Plast Reconstr Surg.

[8] Santana-Cabrera L, Rodríguez-Escot C, Eugenio-Robaina P, Sánchez-Palacios M (2012). Orbital cellulitis and subdural empyema as a complication of dental extraction. Med Intensiva.

[9] Harrison HC (1989). Orbital cellulitis with abscess formation caused by sinusitis. Ann Otol Rhinol Laryngol.

[10] El Mograbi A, Ritter A, Najjar E, Soudry E (2019). Orbital complications of rhinosinusitis in the adult population: analysis of cases presenting to a tertiary medical center over a 13-year period. Ann Otol Rhinol Laryngol.

[11] Elner VM, Demirci H, Nerad JA, Hassan AS (2006). Periocular necrotizing fasciitis with visual loss pathogenesis and treatment. Ophthalmology.

[12] Erickson BP, Lee WW (2015). Orbital cellulitis and subperiosteal abscess: a 5-year outcomes analysis. Orbit.

[13] Gavriel H, Jabarin B, Israel O, Eviatar E (2018). Conservative management for subperiosteal orbital abscess in adults: a 20-year experience. Ann Otol Rhinol Laryngol.

[14] Raponi I, Giovannetti F, Buracchi M (2021). Management of orbital and brain complications of sinusitis: a practical algorithm. J Craniomaxillofac Surg.

